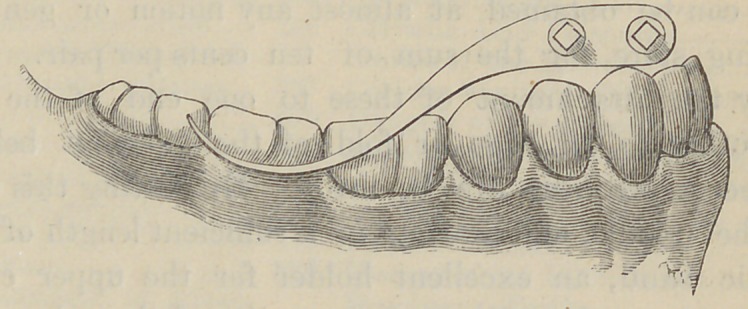# Universal Rubber Dam Screw-Clamp

**Published:** 1881-12

**Authors:** 


### UNIVERSAL RUBBER DAM SCREW-CLAMP.

The accompanying illustrations show the form, size, and application of this instrument.

There are many cases in which it serves a valuable purpose. It can be applied to almost any tooth in the mouth, and in many instances it will afford a support to a frail or tender tooth, that no other appliance, as yet produced, will give. It holds the rubber dam well in place, and gives a rest for the fingers in the various parts of an operation.

When properly applied, it forms a protection for the gums against slipping instruments, better than any other form of clamp. By the use of the screw it can be adjusted to teeth of almost any form or size. The instrument is the device of Dr. E. Parmly Brown, of Flushing, N. Y. The doctor makes it of almost, if not entirely, universal application; and, though others may not accomplish as much with it as he does, yet it will be of great service to every one who uses it intelligently, and to many it will be almost indispensable after reasonable use, and familiarity with it. No operator can afford to be without so valuable an instrument.

## Figures and Tables

**Figure f1:**
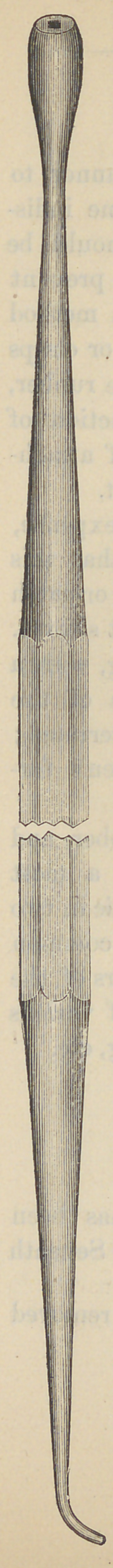


**Figure f2:**
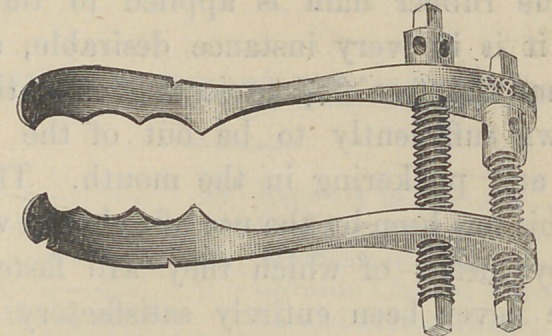


**Figure f3:**